# Nearshore Zone Dynamics Determine Pathway of Organic Carbon From Eroding Permafrost Coasts

**DOI:** 10.1029/2020GL088561

**Published:** 2020-07-31

**Authors:** Dirk Jong, Lisa Bröder, George Tanski, Michael Fritz, Hugues Lantuit, Tommaso Tesi, Negar Haghipour, Timothy I. Eglinton, Jorien E. Vonk

**Affiliations:** ^1^ Department of Earth Sciences Vrije Universiteit Amsterdam Amsterdam The Netherlands; ^2^ Geological Institute Swiss Federal Institute of Technology (ETH) Zürich Switzerland; ^3^ Alfred Wegener Institute Helmholtz Centre for Polar and Marine Research Potsdam Germany; ^4^ Institute for Geosciences University of Potsdam Potsdam Germany; ^5^ CRN Institute of Polar Sciences Bologna Italy

**Keywords:** permafrost, coastal erosion, nearshore zone, particulate organic carbon, carbon cycle, Arctic

## Abstract

Collapse of permafrost coasts delivers large quantities of particulate organic carbon (POC) to Arctic coastal areas. With rapidly changing environmental conditions, sediment and organic carbon (OC) mobilization and transport pathways are also changing. Here, we assess the sources and sinks of POC in the highly dynamic nearshore zone of Herschel Island‐Qikiqtaruk (Yukon, Canada). Our results show that POC concentrations sharply decrease, from 15.9 to 0.3 mg L^−1^, within the first 100–300 m offshore. Simultaneously, radiocarbon ages of POC drop from 16,400 to 3,600 ^14^C years, indicating rapid settling of old permafrost POC to underlying sediments. This suggests that permafrost OC is, apart from a very narrow resuspension zone (<5 m water depth), predominantly deposited in nearshore sediments. While long‐term storage of permafrost OC in marine sediments potentially limits biodegradation and its subsequent release as greenhouse gas, resuspension of fine‐grained, OC‐rich sediments in the nearshore zone potentially enhances OC turnover.

## Key Points

- Suspended particulate organic carbon (POC) sharply decreases in age and concentration within the nearshore zone
- Beyond a narrow resuspension zone, permafrost‐derived OC dominates surface sediments, whereas much younger OC is found in the water column
- Permafrost‐derived POC is rapidly removed from the water column, showing the critical role of nearshore dynamics for OC transport

## 1 Introduction

Northern Hemisphere soils store a large amount of organic carbon (OC), the bulk of which has been frozen in permafrost for millennia (Hugelius et al., 2014; Tamocai et al., 2009; Zimov et al., 2006). These soils present a carbon pool estimated to be almost twice the amount that is currently in the atmosphere (Ciais et al., 2013). Climate warming, particularly enhanced in the north, causes permafrost to warm and thaw, a process that is occurring at the global scale (Biskaborn et al., 2019). Whereas gradual deepening of the seasonally unfrozen layer on land steadily mobilizes permafrost OC, abrupt permafrost thaw and coastal erosion rapidly releases OC into aquatic systems, estimated to be up to 14 Tg OC per year on a panarctic scale (Vonk et al., 2012; Wegner et al., 2015). This flux is in the same order of magnitude as the OC flux from all major Arctic rivers combined (Fritz et al., 2017; McClelland et al., 2016).

Permafrost coastlines account for roughly a third of the Earth's coasts (Lantuit et al., 2012). Permafrost coasts in the Arctic are becoming increasingly vulnerable to erosion due to an extended open water season, higher frequency of storms, and increasing wave fetch and intensity (Günther et al., 2015; Overeem et al., 2011; Overland et al., 2019). Of the Arctic coast, 65% consists of unconsolidated material with a high ice and OC content (Lantuit et al., 2012). These unconsolidated coasts are vulnerable to erosion and abrupt permafrost thaw (Günther et al., 2015; Jones et al., 2018) and the western Canadian Arctic in particular due to its high ice content (Irrgang et al., 2018). Some of the most distinct erosional features in this region are retrogressive thaw slumps (RTS) (Lantuit & Pollard, 2008; Lantz & Kokelj, 2008; Ramage et al., 2017) or block failure along coastal cliffs (Cunliffe et al., 2018; Jones et al., 2009). RTS features have been increasing in number and in spatial extent in this region over the last 60 years (Lantuit & Pollard, 2008; Lantz & Kokelj, 2008; Ramage et al., 2017).

Coastal erosion and abrupt permafrost thaw close to shore releases sediments and OC directly into the nearshore zone, the shallow zone close to the coast that is strongly influenced by waves and longshore currents. Permafrost OC released to the nearshore zone can be (1) mineralized and potentially released as greenhouse gases (GHGs), (2) deposited in nearshore sediments, and/or (3) transported further offshore by waves, currents, and ice bulldozing (Couture et al., 2018; Fritz et al., 2017; Tanski et al., 2019). Mineralization of OC after release and during transport potentially enhances climate warming by producing additional GHG, facilitating a positive “permafrost carbon feedback” to ongoing climate change (Schuur et al., 2015). On the contrary, burial of OC within sediment in the nearshore zone or further offshore may attenuate this feedback loop (Grotheer et al., 2020; Vonk & Gustafsson, 2013). Yet the Arctic nearshore zone is relatively understudied due to logistical constraints, as it is often too shallow to reach with ice‐breaking vessels and too remote to access by land (Fritz et al., 2017).

The bulk of OC released by permafrost coastal erosion is in particulate form (particulate organic carbon, POC), as opposed to dissolved OC (DOC) (Guo et al., 2007; Tanski et al., 2016). Whereas permafrost DOC is often found to be highly susceptible to degradation in the water column (Mann et al., 2015; Vonk et al., 2013), the degradability and transport mechanisms of the more abundant POC fraction is still poorly understood (Couture et al., 2018; Fritz et al., 2017; Tanski et al., 2016). Rapid sedimentation of permafrost POC due to mineral sorption and ballasting might limit the impact of permafrost POC mobilization on climate due to long‐term burial of OC in nearshore and shelf sediments (Grotheer et al., 2020; Hilton et al., 2015; Vonk et al., 2014). However, other studies have documented a large loss of permafrost OC during onshore as well as protracted cross‐shelf transport and could thus be a source of GHG on seasonal to millennial time scales (Bröder et al., 2018; Tanski et al., 2019; Vonk et al., 2012).

We aim to provide new insights into the pathway of POC in the nearshore zone by coupling geochemical and sedimentological properties of permafrost POC in thaw streams, sea water, and marine surface sediments, to better understand the impact of thawing and eroding permafrost coasts on the carbon cycle and climate, and to bridge the gap between the coast and the outer shelf.

## 2 Local Setting

**Figure 1:**
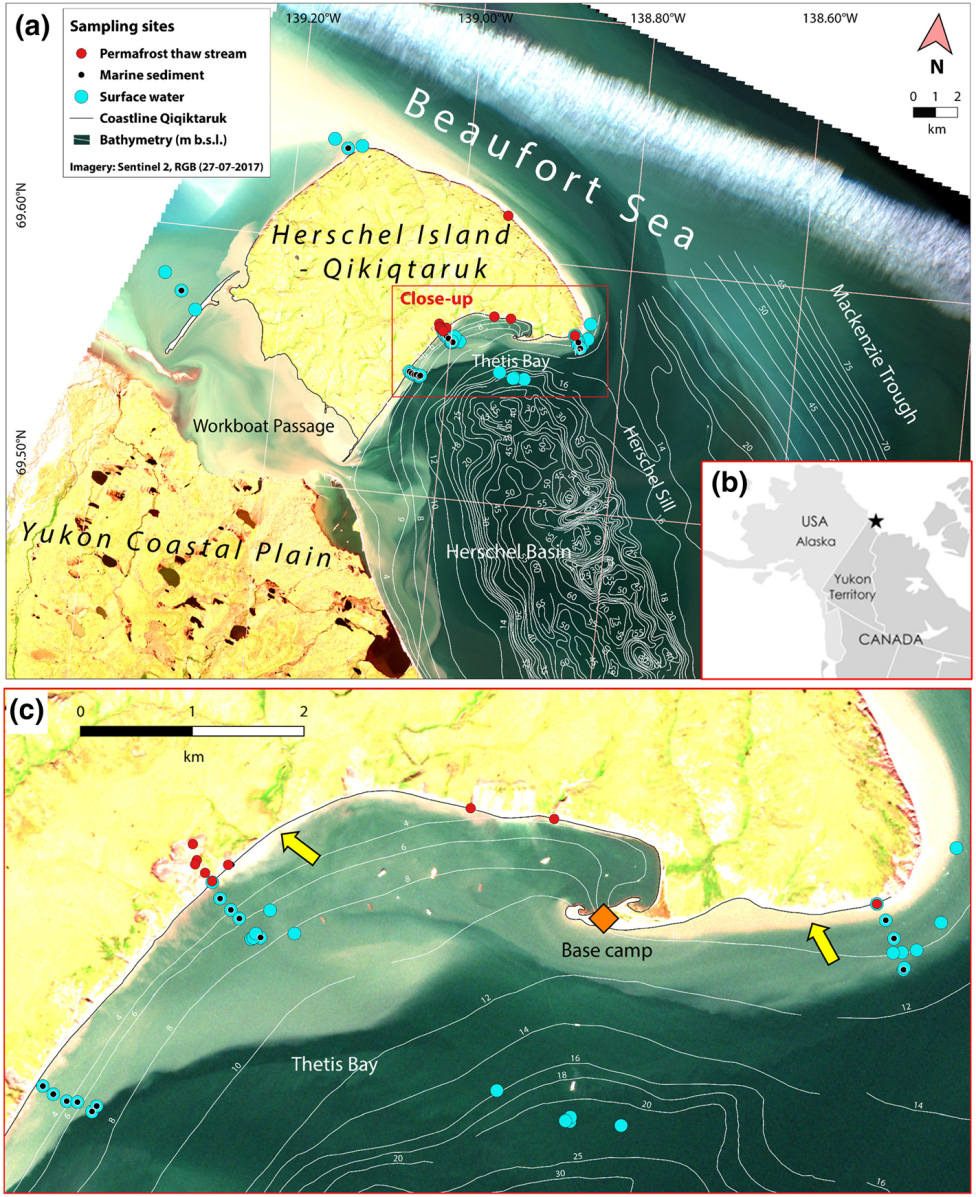
Study area and sample locations. Images: Sentinel 2, RGB. Date: 27 July 2017. (a) Satelite image of Herschel Island‐Qikiqtaruk (Yukon, Canada) and the surrounding Beaufort Sea, with surface water samples shown as blue filled circles, marine sediments as black dots, and permafrost thaw stream samples as red filled circles. (b) Overview map of Alaska and Canada with a black star marking the study area. (c) Close‐up of the main sample area and base camp location. Note the gradient in suspended matter offshore: very turbid sediment plumes within 100–300 m off the coast (highlighted with yellow arrows), fading to less turbid plumes further offshore.

Herschel Island‐Qikiqtaruk is located in the western Canadian Arctic just off the Yukon coast in the Beaufort Sea (N69.60°; W139.00°, Figure 1). The island is an ice‐thrust moraine formed by the Laurentide Ice Sheet during the Late Wisconsin and consists of glacially reworked marine and terrestrial sediments (Fritz et al., 2012; Rampton, 1982). The whole island is composed of ice‐rich continuous permafrost (Couture et al., 2018; Pollard, 1990). The mean coastal erosion rate was on average 0.45 m per year between 1970 and 2000 (Lantuit & Pollard, 2008) but has been increasing to 0.68 m per year for the period 2000–2011 (Obu et al., 2016). However, headwall retreat rates of up to 22 m per year are found at certain RTS features (Obu et al., 2017; Solomon, 2005), and a coastal retreat of 14 m during one summer season was found along low cliffs (Cunliffe et al., 2018). RTS features on the island are highly active and increasing in size, resulting in release of permafrost‐POC via thaw streams into the sea (Ramage et al., 2017; Tanski et al., 2017). During the ice‐free season (June–September), the Mackenzie River outflow causes a brackish upper mixed layer of 5–10 m deep, which reaches the eastern shore of Herschel Island‐Qikiqtaruk (Doxaran et al., 2012; Macdonald & Yu, 2006). The local tidal range is small (±0.5 m); however, storm surges raise the sea water level by several meters (Harper, 1990).

## 3 Methods

In order to trace the pathway of permafrost OC from its source to the nearshore water column and sediments, we collected samples along transects perpendicular to the coastline and at point locations offshore and onshore. A total of 49 locations was visited over a 2‐week period in July–August 2017 at the coast of Herschel Island‐Qikiqtaruk (Figure 1). Twelve of these locations were situated on land at thaw streams, creeks carrying material from RTS's, and other abrupt thaw features. Another 37 sample locations were situated in the nearshore zone, ranging from right at the shoreline to up to 20 m water depth (about 2 km offshore).

Suspended particulate matter (SPM) was sampled at each location from thaw streams or surface water, and surface sediment samples (top 5 cm) were taken with a Van Veen grab sampler. Total OC (TOC, wt.%), POC (mg L^−1^) and total nitrogen (TN, wt.%) were measured, and the C/N ratio and stable carbon isotope (δ^13^C, ‰ Vienna PeeDee Belemnite, VPDB) signal were used as a source indicator. Terrestrial OC generally has a more negative δ^13^C values and higher C/N ratios versus marine OC having a less negative δ^13^C and lower C/N (Meyers, 1994). C/N can also be used as a proxy for carbon mineralization in organic matter, with a lower C/N ratio representing more degraded organic matter (Meyers, 1994; Strauss et al., 2015). Radiocarbon analyses (Δ^14^C) were done on POC and sediment OC and serve as an indicator for the contribution of pre‐aged permafrost OC (e.g., Sánchez‐García et al., 2011; Vonk et al., 2012). In addition, mineral surface area (SA) was measured on sediment samples to characterize sedimentological properties and assess OC‐mineral association. Detailed laboratory and sampling methods can be found in the supporting information.

## 4 Results and Discussion

### 4.1 Distinctive Nearshore Subzones Determine Distribution of OC

**Figure 2:**
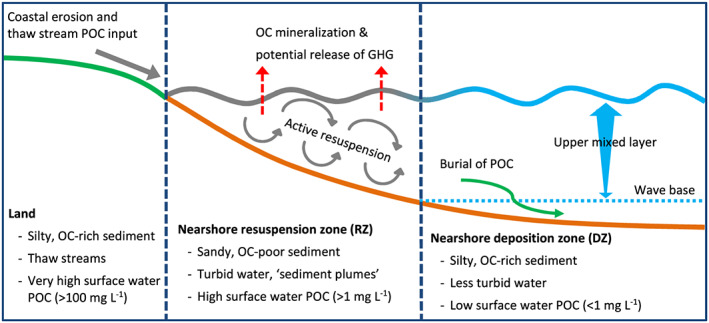
Conceptual model of POC pathways at the coast and in the nearshore zone. The nearshore zone is divided in the resuspension zone (RZ) and the deposition zone (DZ). Suspended matter samples and sediment samples were collected in each zone (for further information see Figure 3). In the upper mixed layer, turbulent mixing by waves and currents is stronger at the surface and decreases with depth (blue arrow). During water column transport, OC can be remineralized and potentially released to the atmosphere as greenhouse gases (GHGs, red arrow) or reach the deposition zone and settle out to the sediment (green arrow).

**Figure 3:**
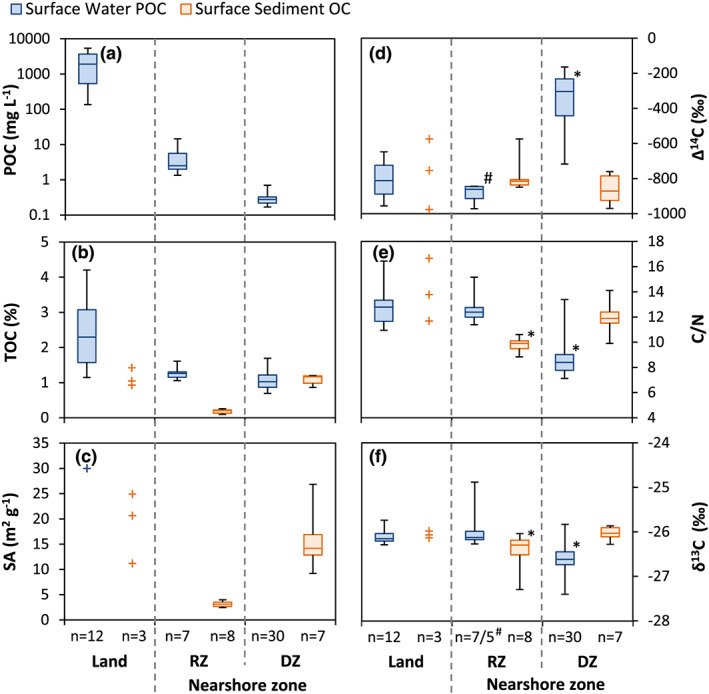
Box‐whisker plots showing the distribution and characteristics of surface water POC (blue boxes) and surface sediment OC (orange boxes) at the coast on land and in the two subzones of the nearshore, the resuspension zone (RZ), and deposition zone (DZ). (a) POC concentration in surface water, note the logarithmic axis. (b) Total organic carbon concentration in % dry weight. (c) Mineral surface area of sediments and one thaw stream sample. (d) Δ^14^C in ‰, 0 ‰ is recent carbon, −1,000‰ is ancient carbon. (e) Carbon to nitrogen (C/N) ratio (mol/mol). (f) δ^13^C in ‰ VPDB. Significantly different OC pools (*p* < 0.01, two‐tailed *t* test) are indicated with an asterisk (*) in panels (d)–(f). Individual measurements are shown with crosses (+) if *n* < 5. ^#^ For the Δ^14^C of RZ POC *n* = 5, as one sample was lost during analyses and one sample was indistinguishable from the background Δ^14^C signal.

Permafrost POC is transported in suspension upon erosion and can subsequently be deposited in nearshore sediments, transported further offshore, or remineralized and released as GHG (Figure 2). Thaw streams and erosion of coastal cliffs deliver highly concentrated SPM directly to the nearshore zone, with a mean POC concentration of 2,200 mg L^−1^ (136–5,350 mg L^−1^; Figure 3a and Table S1 in the supporting information), and a mean OC concentration of the SPM of 2.4 wt.% (1.1–4.2 wt.%; Figure 3b). At the coastline, mud lobes, cliff toe debris, and blocks of permafrost dislodged by coastal erosion were found. These temporary deposits are very fine grained (silt and silty clay), with an OC concentration of 1.2 wt.% (1.0–1.5 wt.%). The fine‐grained, OC‐rich material transported to the nearshore by thaw streams and coastal erosion are prone to rapid removal by waves and brought into suspension. Our observations and measurements indicate that the pathway of this thaw‐mobilized terrestrial material can be delineated into two distinctive nearshore subzones, roughly corresponding to the “upper shoreface” and “lower shoreface” sedimentary environments (Niedoroda et al., 1984). For the purpose of this study, to include water column SPM dynamics, we suggest the terms “nearshore resuspension zone,” and “nearshore deposition zone” (Figure 2).

The “resuspension zone” is defined here as the part of the nearshore zone adjacent to the coastline where sediment is continuously reworked by wave action, resulting in coarse, sandy sediment, and turbid sediment plumes with high SPM concentrations in the water column. This can be seen in seven surface water POC samples from the resuspension zone, which have a significantly higher POC concentration (1.5–15.9 mg L^−1^, mean 4.8 mg L^−1^; Figure 3a and Table S2) than surface water POC samples in the deposition zone (<1.0 mg L^−1^, mean 0.3 mg L^−1^; Figure 3a). The turbid resuspension zone extends approximately 100–300 m offshore in our study area (up to 5–6 m depth), where a sharp boundary separates the resuspension zone from less turbid surface waters, fading further offshore to clearer surface water (Figures 1 and 2). A similarly strong decline in nearshore surface water turbidity was found in the first 100–300 m offshore of Herschel Island‐Qikiqtaruk, indicating rapid settling of material derived from coastal erosion (Klein et al., 2019).

We defined the “deposition zone” as the part of the nearshore zone where fine‐grained sediment and particulates are able to settle out from the water column and are retained in the sediment for a longer period of time. Here, fine‐grained, OC‐rich silts, and clays are found in the sediments, with an average OC concentration of 1.1 wt.% (0.9–1.4 wt.%; Figure 3b) and a relatively high SA of 15.7 m^2^ g^−1^ (9.2–26.8 m^2^ g^−1^; Figure 3c). In contrast, sediments in the resuspension zone are sandy (fine‐medium sand) with a low OC concentration of 0.2 wt.% (0.1–0.3 wt.%; Figure 3b) and have a low SA of 3.1 m^2^ g^−1^ (2.4–4.0 m^2^ g^−1^; Figure 3c), which indicates winnowing of sediment by waves and currents and loss of fine OC‐rich material.

The extent of the resuspension zone and beginning of the deposition zone is determined by the energy of the waves and currents, which depends on many variables, such as wind speed, fetch and direction, and bathymetry, coastline morphology, freshwater inflow, and sea ice conditions. The exact boundary between these zones is therefore never at the exact same location and can be different for the surface water and the sediment (Niedoroda et al., 1984). Resuspension of sediment does substantially increase during (severe) storm events (Hill & Nadeau, 1989) and changes over the course of the seasons (Macdonald et al., 2015). In general, the depth of the resuspension zone can be linked to the upper wind‐mixed layer, the surface water layer that is well mixed by wind, waves, and currents (Dunton et al., 2006). The dense, SPM‐loaded thaw stream water and resuspended nearshore sediment can also be transported along the sediment‐water interface and thus remain undetected in surface water turbidity measurements (Macdonald et al., 2015; O'Brien et al., 2006).

Salinity and temperature measurements along nearshore transects revealed an upper mixed layer of approximately 4 to 6 m depth, with a salinity of 23 and a temperature of 7°C to 10°C (Figure S1). Below this depth, the salinity gradually increased to a value of 29, with temperature decreasing toward 2°C (Figure S1). These observations fit with the general late summer oceanographic setting of the Beaufort Shelf in proximity to the Mackenzie River, with a distinguishable brackish upper mixed layer of 5–10 m depth that can stretch to the eastern coast of Herschel Island‐Qikiqtaruk (Doxaran et al., 2012; Macdonald et al., 1998) and fit well with the range of the resuspension zone.

The shift from high water column POC and low sediment OC to low surface water POC and high sediment OC within the nearshore zone indicates that sediment transport is closely coupled to the distribution of OC. A linear correlation was found between OC concentration and mineral SA (Figure S2; *R* ^2^ = 0.75), suggesting close interaction between organic matter and mineral surfaces (Hedges & Keil, 1995). The OC loading per square meter of sediment is between 0.4 and 1.0 mg OC m^−2^ (Table S3), which is in a similar range as previously reported in marine sediment of the Beaufort Sea (between 0.5 and 1.0 mg OC m^−2^) (Goñi et al., 2005; Hedges & Keil, 1995). Sedimentation of POC due to mineral sorption and ballasting has been suggested elsewhere, increasing the potential for burial of OC in nearshore and shelf sediments (Hilton et al., 2015; Keil et al., 1994; Vonk et al., 2014). The sediment from the beach and the deposition zone (high SA, high OC, and fine, silty sediment) have similar average OC loading of 0.75 ± 0.2 mg OC m^−2^ (*n* = 11), while the low SA, low OC sandy sediment of the resuspension zone has a slightly lower average OC loading of 0.61 ± 0.2 mg OC m^−2^ (*n* = 8). This suggests that material from thaw streams and coastal erosion is transported through the water column and settles to the sediment without significant desorption of OC from mineral particles during transport from land to the deposition zone.

### 4.2 Characteristics of Nearshore POC and Sediment OC

Surface water POC in the deposition zone shows significantly different carbon characteristics than surface water POC in the other two zones, with a mean Δ^14^C value of −349‰ (approximately 3,600 ^14^C years; all ages reported as uncalibrated ^14^C years), ranging from −165‰ to −717‰ (1,400 to 10,100 ^14^C years; Figure 3d), a C/N ratio of 8.7 (7.1 to 13.4; Figure 3e), and δ^13^C value of −26.6‰ (−25.8‰ to −27.4‰; Figure 3f). In sharp contrast, the resuspension zone POC show a much older mean Δ^14^C value of −867‰ (16,400 ^14^C years; Figure 3d). The C/N ratio of the POC samples in the resuspension zone is 12.6 (11.4 to 15.2; Figure 3e), and δ^13^C value of −25.9‰ (−24.9‰ to −26.3‰; Figure 3f). The POC transported to the nearshore zone by thaw streams is very old, with a mean Δ^14^C value of −803‰ (−648‰ to −956‰; 8,300 to 25,000 ^14^C years; Figure 3d). The thaw stream POC C/N ratio is 12.9 (10.9 to 16.4; Figure 3e), and the δ^13^C value is −26.1‰ (−25.8‰ to −27.4‰; Figure 3f), which is similar to the characteristics of the surface water POC in the resuspension zone.

The sediment OC at the coast and in the nearshore zone has a very broad Δ^14^C range, from −544‰ to −978‰ (6,800 to 30,700 ^14^C years; Figure 3d); however, the mean varies little between subzones. The difference in mean Δ^14^C values of sediment on land, in the resuspension zone and the deposition zone is not significant (*p* > 0.1). Resuspension zone sediments do exhibit slightly lower δ^13^C values and C/N ratios. Sediment OC in both zones is significantly older than surface water POC in the deposition zone (*p* < 0.01), yet it is similar to surface water POC in the resuspension zone and thaw stream POC on land. These disparate characteristics suggest rapid settling of old POC upon release to the nearshore zone.

### 4.3 Contrasting Sources of Nearshore Zone POC

Permafrost thaw streams and coastal erosion exert a substantial influence on the marine system in Canadian Arctic coastal regions because of the steady delivery of highly concentrated material to the sea over the summer season (Ramage et al., 2017; Tanski et al., 2016). This dominant terrestrial influence is reflected in the terrestrial δ^13^C‐signature and high C/N ratio of nearshore marine sediments of this study. These results match with results of earlier studies on sediment OC around Herschel Island‐Qikiqtaruk, which show that more than 90% of sediment OC in the nearshore zone is of terrestrial origin (Couture et al., 2018; Grotheer et al., 2020). The range of radiocarbon ages in the SPM and sediment of the nearshore zone reflects the diversity of OC sources in the region. These sources comprise pre‐aged permafrost, the active layer with intermediate radiocarbon age, and contemporary vegetation and marine primary production, which exhibit modern radiocarbon ages. Both the old and the modern pools of terrestrial OC are found exposed at the coast of Herschel Island‐Qikiqtaruk (Fritz et al., 2012), and a recent study suggests that over 50% of sediment OC in Herschel Basin originates from erosion of old coastal permafrost (Grotheer et al., 2020).

The Mackenzie River represents one of the major sources of POC to the Beaufort Sea (McClelland et al., 2016), and lateral transport of Mackenzie River material potentially influences POC and sediment composition in our study area (Grotheer et al., 2020). However, while we do observe the influence of the Mackenzie River water in the salinity and temperature of the surface mixed layer to the east of Herschel Island‐Qikiqtaruk (Figure S1), transport of Mackenzie River SPM this close to the coast of Herschel Island‐Qikiqtaruk is limited (Doxaran et al., 2012; Hill et al., 1991). Instead, it was found that SPM close to the shore of Herschel Island‐Qikiqtaruk is primarily derived from local coastal erosion sources, and that the bulk of it settles out to the nearshore sediments rapidly (Klein et al., 2019). Marine primary production was found to be a minor contributor of OC to nearshore sediments in Herschel Basin (Couture et al., 2018; Grotheer et al., 2020).

While the POC input to the nearshore zone and nearshore sediments are dominated by old, terrestrial material, which is reflected in resuspension zone POC, the contrast with surface water POC in the deposition zone further offshore is striking. The latter exhibits a much younger Δ^14^C value than the surface water POC in the resuspension zone, despite these zones being less than 100–300 m apart. The younger Δ^14^C value of this POC indicates a more dominant influence of modern OC sources, for example, marine primary production or organic debris originating from the active layer and vegetation, instead of old OC stemming from permafrost. While surface water POC in the deposition zone has a terrestrial δ^13^C value of 26.6 ± 0.3‰ (*n* = 30) (Figures 3f and S3), this does not rule out a primary production source at these high latitudes, as primary production in polar waters can produce δ^13^C values between −18‰ and −28‰ (Lamb et al., 2006; Tesi et al., 2017) and C/N ratios between 4 and 10 (Meyers, 1994). While C/N value of 8.7 ± 1.4 (Figures 3e and S3) points toward a primary production source, it could also reflect preferential degradation of younger, more buoyant terrestrial OC (Meyers, 1994; Strauss et al., 2015; Vonk, Sánchez‐García, et al., 2010, Vonk, Van Dongen, & Gustafsson, 2010). Because of this similarity in carbon characteristics, we were unable to distinguish these two end‐members when applying an end‐member mixing model based on our data. Although the exact source of POC in the deposition zone cannot be apportioned, it appears unlikely to be derived from permafrost thaw.

### 4.4 Pathway of Permafrost‐POC Is Determined in the Nearshore Zone

Old, permafrost‐derived POC appears to be initially confined to the water column of the turbid resuspension zone and is not found in the surface water outside of this very narrow zone right in front of the coast, suggesting that this material rapidly settles and accumulates in the underlying sediments. The quick settling and limited water column sediment transport is also observed in a recent study from the same region (Klein et al., 2019). However, in contrast to those findings, we also observe a younger POC pool, which appears to remain in suspension even under more quiescent conditions further offshore. This contrasting sedimentological behavior may be due to preferential settling of the old POC pool as a consequence of its sorption to, and ballasting by mineral particles, while the younger POC pool may not—or to a lower extent—be bound to minerals and therefore remains buoyant over long distances (Vonk, Sánchez‐García, et al., 2010, Vonk, Van Dongen, & Gustafsson, 2010). This interpretation is consistent with our observations of sediment OC and SA, which suggest close interactions between organic matter and mineral surfaces.

The flux of permafrost‐derived POC from coastal erosion is currently equivalent to circum‐Arctic fluvial POC export (Vonk et al., 2012; Wegner et al., 2015). This flux is expected to strongly increase in the near future (Jones et al., 2009; Overland et al., 2019) with anticipated increases in the duration of the open water season and higher storm frequency in a warming Arctic (Günther et al., 2015; Lantuit et al., 2012; Overeem et al., 2011). Moreover, these changes may lead to not only enhanced coastal erosion but also an expansion in the spatial extent of the resuspension zone, further increasing remobilization of permafrost POC sequestered in surface sediments. However, to determine and quantify the long‐term fate of this material, future research should increase its focus on degradation of permafrost‐POC during water column transport in the shallow nearshore zone and its further offshore transport at the sediment‐water interface.

## 5 Conclusions

We find that old POC within the high‐energy resuspension zone originate from coastal erosion and permafrost thaw, while younger POC in surface waters further offshore is derived from marine primary production or contemporary terrestrial organic debris, originating from the active layer and modern vegetation. This suggests rapid and preferential sedimentation of old, mineral‐bound POC derived from coastal permafrost erosion. Close mineral association of OC, together with burial in sediments, may inhibit degradation and limit conversion to GHG. Consequently, these findings suggest that the nearshore zone serves as a potential sink for old, permafrost‐derived POC. However, wave action and currents impact the sea bed, leading to frequent resuspension of nearshore sediment deposits (e.g., during storm events), promoting degradation of associated OC to GHG. This implies that a relatively narrow coastal zone may exert a strong influence on the fate of permafrost POC mobilized from Arctic coastal settings. We provide insight in the pathway of permafrost‐POC in the water column of the dynamic nearshore zone and its interactions with the underlying sediment. Furthermore, we hypothesize that the long‐term fate of permafrost‐POC is closely linked to the dynamic environment of the nearshore zone.

## Supporting information



Supporting Information S1

## Data Availability

Samples and data used in this study are available in the supporting information and can be accessed at PANGAEA (https://doi.pangaea.de/10.1594/PANGAEA.913721).
