# Supplementary material for: Nearshore Zone Dynamics Determine Pathway of Organic Carbon From Eroding Permafrost Coasts
Source: Geophys Res Lett. 2020 Jul 31;47(15):e2020GL088561. doi: 10.1029/2020GL088561 (PMC7507779; doi:10.1029/2020GL088561)
Supplement: Supplementary file 1 — Supporting Information S1 [file GRL-47-e2020GL088561-s001.pdf]

AGU PUBLICATIONS

Geophysical Research Letters

Supporting Information for

Nearshore zone dynamics determine pathway of organic carbon from eroding permafrost coasts

Dirk Jong[1*], Lisa Bröder[1,2], George Tanski[1,3], Michael Fritz[3], Hugues Lantuit[3], Tommaso Tesi[4], Negar Haghipour[2], Timothy I. Eglinton[2], Jorien E. Vonk[1*]

[1]Vrije Universiteit Amsterdam, The Netherlands. [2]Swiss Federal Institute of Technology (ETH), Zürich, Switzerland. [3]Alfred Wegener Institute, Potsdam, Germany. [4]CRN, Institute of Polar Sciences, Bologna, Italy.

*Corresponding author: Dirk Jong (d.j.jong@vu.nl), Jorien E. Vonk (j.e.vonk@vu.nl)

Contents of this file

Supplementary methods
Figure S1 – S3
Table S1 – S3
References

Introduction

Collapse of permafrost coasts delivers large quantities of particulate organic carbon (POC) to arctic coastal areas. The objective of this study is to assess the pathway of POC derived from thawing and eroding permafrost coasts in the nearshore zone, to better understand its impact on the carbon cycle and climate. We collected suspended particulate matter and sediment samples at the coastline and in the nearshore zone of Herschel Island - Qikiqtaruk (N 69.60°; W 139.00°) during a two-week period in July-August 2017. Locations were labelled A - H clockwise around the island, starting at the NE corner (Collinson Head). Samples were taken in transects perpendicular to the coast, at point locations offshore ('Monitoring point 1 - 3'), and from 'thaw streams', creeks carrying material from retrogressive thaw slumps and other abrupt thaw features, at various locations around the island. A total of 49 locations were visited; 12 at thaw streams, and 37 in the nearshore zone ranging from right at the shoreline to up to 20 m water depth (about 2 km offshore). Conductivity, temperature and depth (CTD) of the water column was measured using a CastAway-CTD® to account for the oceanographic and hydrodynamic setting at the time of sampling (Figure S1). The supporting information contains the detailed laboratory methods and the data tables used in this study.

Supplement methods

Sample preparation

At each sampling location, surface water or thaw stream water was sampled for suspended particulate matter (SPM) using 3 - 5 L LDPE bags or HDPE Nalgene bottles. The sampled water was filtered through pre-combusted (400 °C, 12h) and pre-weighed glass fiber filters (GF/F, Whatman, 0.7µm pore size) using a Nalgene polycarbonate filter tower. Surface sediment (approximately 0 – 5 cm) was sampled using a stainless-steel Van Veen grab sampler and stored in Whirl-Pak® bags. All samples were immediately stored at -20 °C, and kept frozen during transport from Herschel Island – Qikiqtaruk to the Vrije Universiteit Amsterdam, where the samples were further processed. All samples were freeze-dried, and sediments were homogenized and split for further analyses.

Organic carbon and isotope analyses

SPM concentration was determined by weighing the GF/F filters. Then, the filters and sediment samples were subsampled for elemental and sedimentological analyses. Subsamples for bulk elemental (C, N) and carbon isotope ($\delta^{13}C$, $\Delta^{14}C$) analyses of SPM samples were punched out of the 47mm GF/F filters, weighed and placed in pre-combusted silver capsules. Sediment subsamples for these analyses were homogenized and crushed in an agate mortar and weighed into pre-combusted silver capsules. Inorganic C was removed from the filter and sediment subsamples by fumigation in a desiccator with 37% HCl at 60 °C for 72h (Komada et al., 2008). Afterwards, samples were dried over NaOH at 60 °C for 48h to neutralize the acid. The silver capsules were wrapped in tin, and analyzed for OC, TN and $\delta^{13}C$ by elemental analyzer - isotope ratio mass spectrometer (EA-IRMS) at the University of California's Stable Isotope Facility (Davis, United States) and at the National Research Council Institute of Polar Sciences (Bologna, Italy). The $\delta^{13}C$ is reported in ‰ relative to Vienna Pee Dee Belemnite (VPDB). Radiocarbon analyses ($\Delta^{14}C$) were carried out using a MICADAS accelerator mass spectrometer (AMS) at the Laboratory of Ion Beam Physics of the Swiss Federal Institute of Technology (Zürich, Switzerland), using the method described in McIntyre et al. (2017). The $\Delta^{14}C$ results are corrected for constant background contamination using the method described in Haghipour et al. (2018).

Sedimentological analyses

For mineral surface area (SA) measurements, subsamples of about 1.5 g sediment were combusted at 450 °C for 12 h to remove OC, rinsed twice with MilliQ to remove salt and ashes, and freeze dried. Directly prior to analysis, the samples were degassed at 300 °C under vacuum and measured with a Quantachrome Nova 4200e, using the 6-point Brunauer–Emmett–Teller method (Brunauer et al., 1938). SA measurements were checked against two certified reference materials (of 5.41 and 27.46 m^2 g^{-1}) and show an accuracy of 4%. Grain size was categorized in sand (medium/fine), silty sand, silt, and silty clay after visual inspection, and grain size distribution was measured on a selection of representative samples by laser diffraction on a Sympactec Helos Laser KR.

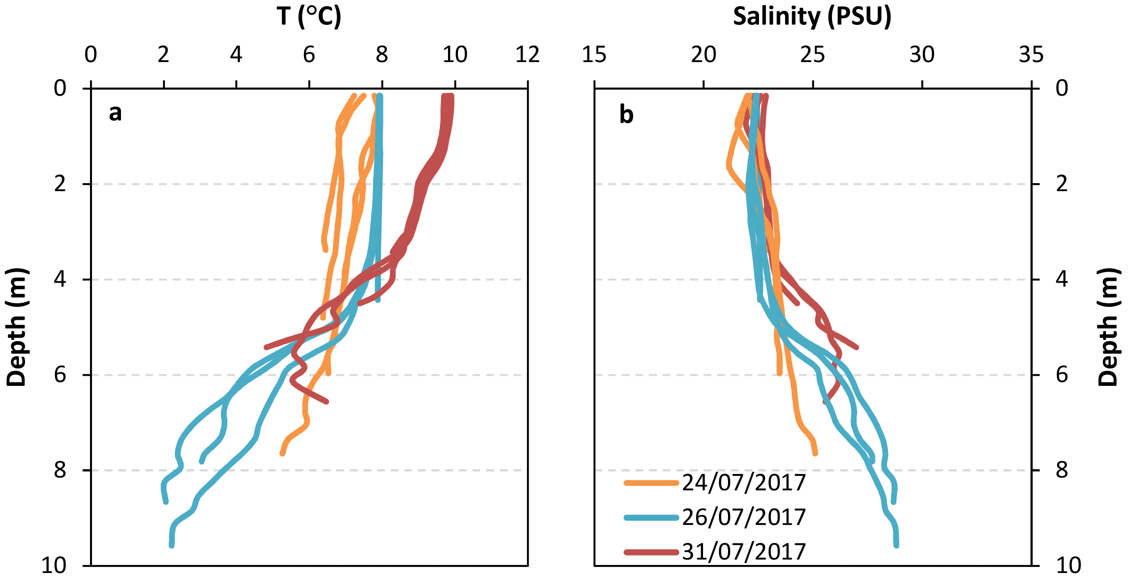

Figure S1. a. Temperature and **b.** salinity data collected at various water depths on three sampling days shows an upper mixed layer of 4 – 6 m deep. CTD measuring locations are: D2 – D5 (24 July 2017), B1 – B4 (26 July 2017), E2 – E5 (31 July 2017), coordinates can be found in Data table S1.

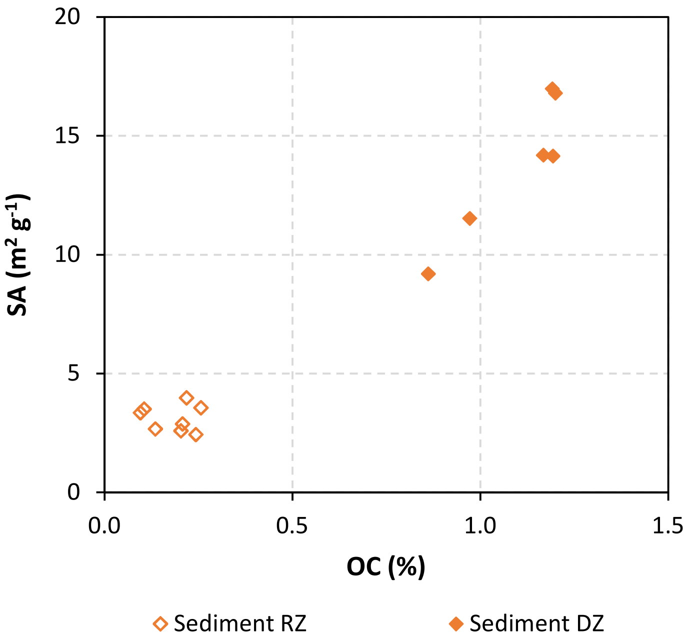

Figure S2. Mineral surface area (SA, $m^2\ g^{-1}$) versus organic carbon (OC, weight %) of sediment in the resuspension zone (RZ) and deposition zone (DZ).

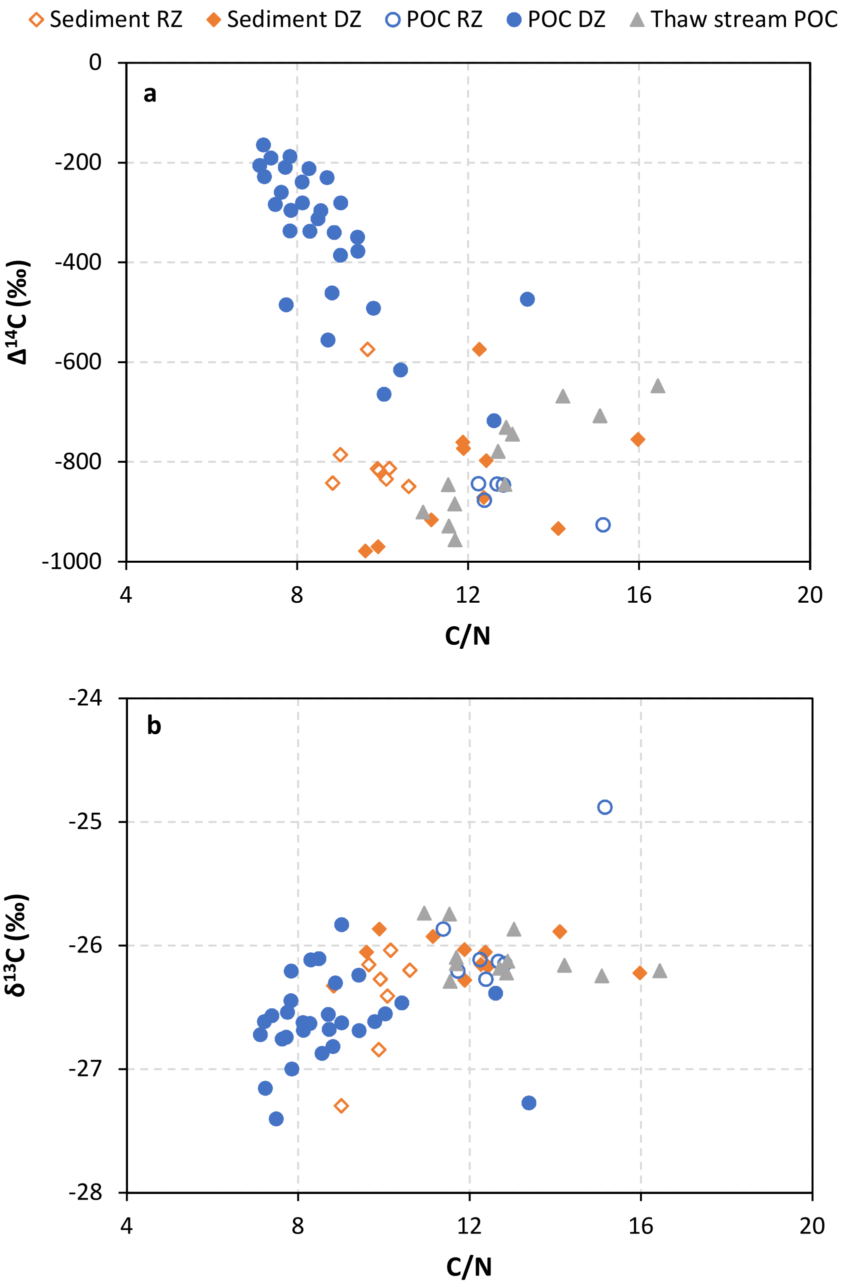

Figure S3. a. $\Delta^{14}C$ (‰) ratio versus C/N (mol/mol) ratio of sediment and suspended particulate organic carbon (POC) in the resuspension zone (RZ) and deposition zone (DZ), and of thaw stream POC. **b.** $\delta^{13}C$ (‰) versus C/N (mol/mol) ratio of the same samples.

Table S1. Biogeochemical data of thaw stream suspended particulate matter.

Sample name	Date	Location		SPM	POC	OC	TN	C/N	δ¹³C	Δ¹⁴C	¹⁴C age	¹⁴C raw data		ETH lab number
YC17_xx		Latitude	Longitude	(g L^{-1})	(g L^{-1})	(wt.%)	(wt.%)	(mol)	(‰)	(‰)	(Kyr.)	Fm	±	
D_TS_00	24/07/2017	69.56799	-139.01003	75.7	1.91	2.53	0.23	12.7	-26.2	-778.8	12.1	0.223	0.010	88031.1.1
B_TS_01	26/07/2017	69.57159	-138.85753	68.7	1.09	1.59	0.17	10.9	-25.7	-900.1	18.4	0.101	0.011	88032.1.1
B_TS_02	26/07/2017	69.57159	-138.85753	50.8	0.58	1.15	0.12	11.5	-25.7	-845.1	14.9	0.156	0.007	91245.1.1
A_TS_01	26/07/2017	69.61740	-138.94837	25.3	0.39	1.52	0.15	11.7	-26.1	-883.9	17.2	0.117	0.008	91264.1.1
C_TS_01	27/07/2017	69.57599	-138.95264	204.6	3.49	1.71	0.15	12.9	-26.2	-844.7	14.9	0.157	0.009	88038.1.1
D_TS_01	31/07/2017	69.56944	-139.00676	10.7	0.14	1.32	0.13	11.7	-26.1	-955.9	25.0	0.044	0.007	91266.1.1
D_TS_02	31/07/2017	69.57082	-139.01533	71.6	1.91	2.66	0.24	13.0	-25.9	-744.2	10.9	0.258	0.009	88033.1.1
D_TS_03	31/07/2017	69.56956	-139.01402	68.5	2.13	3.11	0.26	14.2	-26.2	-667.8	8.8	0.335	0.009	88034.1.1
D_TS_04	31/07/2017	69.56921	-139.01432	152.1	4.65	3.06	0.28	12.9	-26.1	-730.2	10.5	0.272	0.009	88035.1.1
D_TS_05	31/07/2017	69.56860	-139.01183	128.4	5.39	4.20	0.30	16.4	-26.2	-647.6	8.3	0.355	0.009	88036.1.1
D_TS_06	31/07/2017	69.56799	-139.01003	136.1	4.32	3.17	0.25	15.1	-26.2	-707.0	9.8	0.295	0.008	88037.1.1
C_TS_02	31/07/2017	69.57581	-138.93328	6.6	0.14	2.06	0.21	11.5	-26.3	-928.2	21.1	0.072	0.007	91268.1.1

Table S2. Biogeochemical data of nearshore surface water suspended particulate matter.

Sample name	Date	Location		Water depth	SPM	POC	OC	TN	C/N	δ¹³C	Δ¹⁴C	¹⁴C age	¹⁴C raw data		ETH lab number
YC17_xx_SW		Latitude	Longitude	(m)	(mg L^{-1})	(mg L^{-1})	(wt.%)	(wt.%)	(mol)	(‰)	(‰)	(Kyr)	Fm	±	
MP1	21/07/2017	69.56368	-138.99973	7.2	23.5	0.32	1.34	0.17	9.4	-26.7	-377	3.7	0.628	0.028	91269.1.1
MP2	21/07/2017	69.55164	-138.92266	20.7	20.1	0.28	1.41	0.22	7.5	-27.4	-284	2.6	0.722	0.039	91271.1.1
MP3	21/07/2017	69.56821	-138.84739	9.5	29.3	0.35	1.19	0.16	8.7	-26.7	-555	6.4	0.448	0.032	91272.1.1
MP1	23/07/2017	69.56447	-138.98999	8.3	23.8	0.33	1.39	0.21	7.9	-27.0	-296	2.8	0.710	0.032	91273.1.1
MP2	23/07/2017	69.55197	-138.92265	16.9	45.5	0.56	1.22	0.14	10.4	-26.5	-615	7.6	0.388	0.027	91274.1.1
MP3	23/07/2017	69.56792	-138.85068	9.5	47.8	0.66	1.37	0.16	10.0	-26.6	-664	8.7	0.338	0.022	91275.1.1
D1*	24/07/2017	69.56790	-139.00999	0.6	383.4	6.17	1.61	0.12	15.2	-24.9	-926	20.8	0.075	0.031	91235.1.1
D2*	24/07/2017	69.56666	-139.00779	3.4	192.3	2.42	1.26	0.11	12.8	-26.1	-846	15.0	0.155	0.018	91236.1.1
D3*	24/07/2017	69.56584	-139.00508	4.8	111.2	1.34	1.21	0.12	11.7	-26.2					91237.1.1
D4	24/07/2017	69.56521	-139.00293	6.0	58.5	0.70	1.19	0.11	12.6	-26.4	-717	10.1	0.285	0.033	91238.1.1
D5	24/07/2017	69.56386	-138.99764	7.7	34.8	0.45	1.30	0.15	9.8	-26.6	-492	5.4	0.512	0.026	91239.1.1

MP1	25/07/2017	69.56613	-138.99609	6.5	21.4	0.31	1.45	0.23	7.2	-26.6	-165	1.4	0.842	0.015	91276.1.1
MP2	25/07/2017	69.55174	-138.91087	19.5	19.1	0.22	1.17	0.19	7.2	-27.2	-228	2.0	0.778	0.035	91279.1.1
MP3	25/07/2017	69.57674	-138.84063	9.4	27.9	0.47	1.69	0.15	13.4	-27.3	-474	5.1	0.531	0.022	91278.1.1
MP3*	25/07/2017	69.57060	-138.84243	7.1	147.3	1.56	1.06	0.10	12.2	-26.1	-843	14.8	0.158	0.021	91277.1.1
Bo*	26/07/2017	69.57159	-138.85753	0.2	1119.4	14.60	1.30	0.13	11.4	-25.9			0.028	0.032	91244.1.1
B1	26/07/2017	69.57035	-138.85513	4.4	26.2	0.27	1.04	0.14	8.7	-26.6	-230	2.0	0.776	0.042	91240.1.1
B2	26/07/2017	69.56895	-138.85282	7.8	25.9	0.29	1.12	0.17	7.7	-26.7	-210	1.8	0.797	0.044	91241.1.1
B3	26/07/2017	69.56781	-138.85269	8.7	28.6	0.29	1.01	0.14	8.3	-26.6	-212	1.8	0.794	0.047	91242.1.1
B4	26/07/2017	69.56653	-138.84993	9.6	31.6	0.31	0.98	0.14	8.1	-26.6	-239	2.1	0.768	0.043	91243.1.1
MP1	28/07/2017	69.56378	-138.99930	7.0	29.3	0.27	0.91	0.14	7.8	-26.5	-485	5.3	0.519	0.040	91281.1.1
MP2	28/07/2017	69.55351	-138.94008	15.8	24.3	0.19	0.80	0.11	8.1	-26.7	-280	2.6	0.725	0.057	91282.1.1
MP3	28/07/2017	69.56669	-138.84981	9.5	30.6	0.37	1.19	0.16	8.8	-26.8	-461	4.9	0.543	0.029	88045.1.1
G1	29/07/2017	69.56618	-139.29859	3.8	30.8	0.24	0.77	0.10	9.4	-26.2	-350	3.4	0.656	0.056	91251.1.1
G2	29/07/2017	69.57329	-139.31692	8.3	28.8	0.29	1.01	0.13	9.0	-25.8	-386	3.8	0.619	0.040	91253.1.1
G3	29/07/2017	69.58007	-139.33794	11.1	26.8	0.26	0.98	0.13	8.9	-26.3	-340	3.3	0.665	0.043	91246.1.1
H1*	29/07/2017	69.55013	-139.04387	4.0	228.8	2.51	1.10	0.10	12.7	-26.1	-844	14.9	0.157	0.020	91252.1.1
H2	29/07/2017	69.63814	-139.14164	7.8	26.5	0.26	0.99	0.14	8.3	-26.1	-338	3.2	0.668	0.036	91249.1.1
H3	29/07/2017	69.64138	-139.15801	20.0	24.4	0.17	0.69	0.10	8.5	-26.1	-313	2.9	0.693	0.065	91248.1.1
E1*	31/07/2017	69.54956	-139.04129	1.8	392.1	5.10	1.30	0.12	12.4	-26.3	-876	16.7	0.125	0.010	91258.1.1
E2	31/07/2017	69.54911	-139.03806	3.4	22.0	0.23	1.06	0.17	7.4	-26.6	-191	1.6	0.816	0.031	91254.1.1
E3	31/07/2017	69.54913	-139.03558	4.5	21.9	0.22	0.98	0.16	7.1	-26.7	-206	1.8	0.801	0.052	91255.1.1
E4	31/07/2017	69.54849	-139.03204	5.4	22.9	0.18	0.80	0.12	7.6	-26.8	-260	2.3	0.746	0.049	91256.1.1
E5	31/07/2017	69.54897	-139.03110	6.6	22.4	0.19	0.85	0.13	7.8	-26.4	-188	1.6	0.819	0.053	91257.1.1
MP1	02/08/2017	69.63968	-139.12563	7.4	22.6	0.18	0.81	0.11	8.6	-26.9	-296	2.8	0.709	0.052	88046.1.1
MP2	02/08/2017	69.56413	-138.99882	19.9	25.2	0.19	0.76	0.10	9.0	-26.6	-281	2.6	0.725	0.061	88047.1.1
MP3	02/08/2017	69.55166	-138.92369	9.9	22.6	0.19	0.82	0.12	7.8	-26.2	-337	3.2	0.668	0.039	88048.1.1

* Considered 'Nearshore resuspension zone' samples due to high SPM (> 100 mg L^{-1}) and high POC (>1.0 mg L^{-1}) concentration.
In red: Too low ^{14}C count for accurate dating, use the reported Fm, $\Delta^{14}C$ value and ^{14}C age with caution.

Table S3. Biogeochemical and sedimentological data of nearshore surface sediment.

Sample name	Date	Location		Water depth	OC	TN	C/N	$\delta^{13}C$	SA	OC loading	Grain size description	Mean grain size	$\Delta^{14}C$	^{14}C age	^{14}C raw data		ETH lab number
YC17_xx_SED		Latitude	Longitude	(m)	(wt.%)	(wt.%)	(mol)	(‰)	(m2 g^{-1})	(mg OC m^{-2})		(µm)	(‰)	(Kyr.)	Fm	±	
D1#	24/07/2017	69.56790	-139.00999	0.6	1.07	0.08	16.0	-26.2	10.5	1.02	Silt	60	-755	11.2	0.247	0.004	88008.1.1
D2	24/07/2017	69.56666	-139.00779	3.4	0.26	0.03	10.6	-26.2	3.6	0.72	Fine sand	124	-849	15.1	0.152	0.003	88009.1.1
D3	24/07/2017	69.56584	-139.00508	4.8	0.22	0.03	9.9	-26.3	4.0	0.55	Fine sand	144	-817	13.6	0.184	0.003	88010.1.1
D4*	24/07/2017	69.56521	-139.00293	6.0	0.97	0.09	12.4	-26.2	11.5	0.84	Silt/fine sand	82	-797	12.8	0.204	0.003	88011.1.1
D5*	24/07/2017	69.56386	-138.99764	7.7	1.19	0.12	11.9	-26.0	14.2	0.84	Silty clay	10	-760	11.4	0.242	0.004	88012.1.1
B0#	26/07/2017	69.57159	-138.85753	0.0	0.95	0.12	9.6	-26.1	25.2	0.38	Silt		-978	30.7	0.022	0.001	88013.1.1
B1	26/07/2017	69.57035	-138.85513	4.4	0.10	0.01	9.0	-27.3	3.4	0.29	Medium/fine sand		-785	12.3	0.216	0.004	88014.1.1
B2*	26/07/2017	69.56895	-138.85282	7.8	1.20	0.11	12.4	-26.1	16.8	0.71	Silt		-871	16.4	0.130	0.003	88015.1.1
B4	26/07/2017	69.56653	-138.84993	9.6	1.19	0.12	11.1	-25.9	17.0	0.70	Silt		-916	19.8	0.085	0.002	88016.1.1
H1*	29/07/2017	69.55013	-139.04387	4.0	1.17	0.10	14.1	-25.9	14.2	0.82	Silt/fine sand		-933	21.7	0.067	0.002	88022.1.1
H2*	29/07/2017	69.63814	-139.14164	7.8	0.99	0.12	9.9	-25.9	26.8	0.37	Silt		-970	28.1	0.030	0.002	88023.1.1
G2	29/07/2017	69.57329	-139.31692	8.3	0.11	0.01	9.6	-26.2	3.5	0.30	Medium/fine sand		-574	6.8	0.429	0.005	88024.1.1
E1	31/07/2017	69.54956	-139.04129	1.8	0.14	0.02	8.8	-26.3	2.7	0.51	Medium sand	247	-842	14.8	0.159	0.003	88017.1.1
E2	31/07/2017	69.54911	-139.03806	3.4	0.24	0.03	10.1	-26.4	2.4	0.99	Fine sand	93	-834	14.4	0.167	0.003	88018.1.1
E3	31/07/2017	69.54913	-139.03558	4.5	0.20	0.02	9.9	-26.8	2.6	0.78	Fine sand	103	-813	13.4	0.188	0.003	88019.1.1
E4	31/07/2017	69.54849	-139.03204	5.4	0.21	0.02	10.2	-26.0	2.9	0.72	Fine sand	107	-813	13.4	0.189	0.003	88020.1.1
E5*	31/07/2017	69.54897	-139.03110	6.6	0.86	0.08	11.9	-26.3	9.2	0.94	Silt	34	-773	11.8	0.229	0.004	88021.1.1
D_ML#	31/07/2017	69.56949	-139.00596	0.0	1.45	0.14	12.3	-26.2	20.7	0.70	Silty clay	7	-575	6.8	0.227	0.003	88025.1.1

* Considered 'Nearshore deposition zone' samples due to deposition of fine sediment (silt and clay).
# 'Beach deposits', freshly deposited terrestrial sediment at limited depth or on the beach.

References

Brunauer, S., Emmett, P. H., & Teller, E. (1938). Adsorption of Gases in Multimolecular Layers. *Journal of the American Chemical Society*, *60*(2), 309–319. https://doi.org/10.1021/ja01269a023

Haghipour, N., Ausin, B., Usman, M. O., Ishikawa, N., Wacker, L., Welte, C., … Eglinton, T. I. (2018). Compound-Specific Radiocarbon Analysis by Elemental Analyzer–Accelerator Mass Spectrometry: Precision and Limitations. *Analytical Chemistry*, *91*, 2042–2049. research-article. https://doi.org/10.1021/acs.analchem.8b04491

Komada, T., Anderson, M. R., & Dorfmeier, C. L. (2008). Carbonate removal from coastal sediments for the determination of organic carbon and its isotopic signatures, d13C and D14C: comparison of fumigation and direct acidification by hydrochloric acid. *Limnology and Oceanography: Methods*, *6*, 254–262. https://doi.org/10.4319/lom.2008.6.254

McIntyre, C. P., Wacker, L., Haghipour, N., Blattmann, T. M., Fahrni, S., Usman, M., … Synal, H. A. (2017). Online 13C and 14C Gas Measurements by EA-IRMS-AMS at ETH Zürich. *Radiocarbon*, *59*(3), 893–903. https://doi.org/10.1017/RDC.2016.68
